# Efficient PbS Quantum Dot Solar Cells with Both Mg-Doped ZnO Window Layer and ZnO Nanocrystal Interface Passivation Layer

**DOI:** 10.3390/nano11010219

**Published:** 2021-01-15

**Authors:** Hao Ren, Ao Xu, Yiyang Pan, Donghuan Qin, Lintao Hou, Dan Wang

**Affiliations:** 1 State Key Laboratory of Luminescent Materials & Devices, Institute of Polymer Optoelectronic Materials & Devices, South China University of Technology, Guangzhou 510640, China; renhns@foxmail.com (H.R.); m13856403915@163.com (A.X.); 201730321247@mail.scut.edu.cn (Y.P.); wangdan@scut.edu.cn (D.W.); 2 Guangdong Provincial Key Laboratory of Optical Fiber Sensing and Communications, Guangzhou Key Laboratory of Vacuum Coating Technologies and New Energy Materials, Siyuan Laboratory, Department of Physics, Jinan University, Guangzhou 510632, China

**Keywords:** Mg-doped ZnO, PbS, quantum dot, solar cells

## Abstract

In this paper, a Mg-doped ZnO (MZO) thin film is prepared by a simple solution process under ambient conditions and is used as the window layer for PbS solar cells due to a wide n-type bandgap. Moreover, a thin layer of ZnO nanocrystals (NCs) was deposited on the MZO to reduce carrier recombination at the interface for inverted PbS quantum dot solar cells with the configuration Indium Tin Oxides (ITO)/MZO/ZnO NC (w/o)/PbS/Au. The effect of film thickness and annealing temperature of MZO and ZnO NC on the performance of PbS quantum dot solar cells was investigated in detail. It was found that without the ZnO NC thin layer, the highest power conversion efficiency(PCE) of 5.52% was obtained in the case of a device with an MZO thickness of 50 nm. When a thin layer of ZnO NC was introduced between MZO and PbS quantum dot film, the PCE of the champion device was greatly improved to 7.06% due to the decreased interface recombination. The usage of the MZO buffer layer along with the ZnO NC interface passivation technique is expected to further improve the performance of quantum dot solar cells.

## 1. Introduction

PbS colloidal quantum dot (CQD) solar cells have been rapidly developed in recent years with a certified power conversion efficiency (PCE) up to 13% in an optimal device configuration [1,2]. Compared to traditional photovoltaic technology, PbS CQD solar cells show great potential applications in energy products in view of their low cost, low temperature, and simple solution processing techniques [3,4,5,6,7,8,9,10]. More importantly, the bandgap of PbS CQDs can be easily controlled by varying the size of the CQDs, which enables efficient harvesting of a broad light spectrum from the visible to infrared region. Thus, the fabrication of tandem cells can be easily achieved based on a single material [11,12,13].

The significant performance improvement of PbS CQD solar cells is mainly attributed to the developing ligand exchange technique, interface engineering, and optimized device architecture. It is well known that PbS CQDs are usually capped by insulating ligands, such as oleic acid and oleic amine, during the synthesis processes. These insulating ligands will later become defects and carrier traps in the photovoltaic devices, which are mainly responsible for the low device performance [14,15]. Approaches to replace the insulating ligands must address the issues of the Pb^2+^ oxidation and the shortening of the distance between PbS CQDs. In previous work, 1,2-Ethanedithiol (EDT) or 3-Mercaptopropionic acid was selected as a short bidentate ligand to replace the insulating ligands during the device fabrication process and showed promising outcomes in reducing interparticle spacing and improving device performance [16,17,18]. Later, people found that the hydroxyl (OH) groups attached to (111) facets of PbS nanocrystals will increase the trap states in the CQDs and limit device performance [19,20,21]. By combining metal halide and organic ligands, the density of dangling bonds and trap states can be reduced effectively and result in higher PCE [22,23,24,25]. Unfortunately, there are still some residual OH groups left behind during the ligand exchange process, which further impede the improvement of CQD solar cells. After exploring these challenges for several years, researchers have found some ways to overcome this difficulty. For instance, by adding PbBr_2_ during the synthesis process, the surface of PbS CQDs will reconstruct and the number of OH surface groups will be significantly reduced, which could improve the device PCE to a high level of 12.5% [26].

On the other hand, the electron transfer layers (ETLs) also have a significant effect on the CQD solar cells. Metal oxides, like titanium dioxide (TiO_2_) and zinc oxide (ZnO), are often used as the ETL for PbS CQD solar cells with inverted device architecture [27,28,29,30]. Because of their excellent electron transport properties and simple solution processing techniques, these metal oxides are promising candidates for ETLs in low-cost, efficient CQD solar cells. To improve the CQD solar cell performance, it is essential to increase the carrier mobility of ETLs and construct a preferable band alignment with the active layer. One path to control the electronic properties of ETLs is introducing metal (such as Al, Mg, Sb, etc.) salt into a metal oxide precursor before the ETL film deposition. For example, Liu et al. [31] reported efficient PbS CQD solar cells using Zr-doped TiO_2_ electron-acceptor materials. By changing the doping density, the band structure of the TiO_2_ thin film was modified to enhance charge separation efficiency at the CQD/TiO_2_ interface. In the case of ZnO ETLs, potential doping elements include indium, tin, aluminum, lithium, and other organic dopants [32,33,34]. Introducing double ETLs is another way to improve the carrier separation efficiency between ETLs and the PbS CQD active layer. Eisner et al. [35] adopted In_2_O_3_ (thin layer)/ZnO as a double ETL for quantum dots or organic solar cells. Their devices showed enhanced electron mobility and a tunable Fermi level, which resulted in relatively low series resistance and improved PCE. In our previous work [36], we found that the performance of CdTe nanocrystal (NC) solar cells with an inverted structure of FTO/TiO_2_/CdTe/Au can be largely improved by inserting a thin layer of solution-processed CdS NC film between the CdTe NC and TiO_2_ due to the optimized band alignment and p-n junction quality. The light absorption of the ETLs, however, might also negatively influence the device performance. Hu et al. [37] replaced the ZnO with a Mg-doped ZnO (MZO) layer to broaden the band gap of an ETL to reduce its parasitic absorption. By this method, more light is permitted to go through the ETL and is absorbed by the PbS CQD active layer.

Herein, we present a novel solution processed PbS CQD solar cell which adopts MZO/ZnO NC thin films as double ETLs. By adjusting the deposition conditions of the ZnO NC thin film, behaviors of the MZO/PbS interface are well controlled and a more compact nanocrystalline PbS film could be formed. Various characterizations were performed to inspect the surface morphology of the MZO/ZnO NC thin film for illustrating how these double ETLs help modify the MZO/PbS interface. By introducing the double ETL strategy into solution processed PbS CQD solar cells, photovoltaic devices with an inverted structure of the ITO/MZO/ZnO NC film (*w/o*)/PbS CQDs/Au were fabricated. As the interface between the ETL and PbS CQD films was well controlled, traps at the interface are effectively eliminated and the device performance is greatly improved, which exhibited a high PCE of 7.09%, while the value of the device without the ZnO NC film was 5.52%. In conclusion, devices with double ETLs show almost 30% improvements compared to the control device without the ZnO NC layer. In addition, the results of constant measurement over 30 days indicated that these PbS CQD solar cells are extraordinarily stable under ambient preservation conditions. The simple environmentally friendly fabrication process surely makes the PbS CQD photovoltaic device a promising candidate for low-cost solar cell applications.

## 2. Experiment Procedure

Materials: oleic acid (90%), PbO (99.999%), bis(trimethylsilyl)sulfide ((TMS)_2_S, 98%), octadecene (ODE, 90%), zinc acetate dehydrate, 2-methoxyethanol (99.8%), ethanolamine, magnesium acetate tetrahydrate (99%), ethanolamine (99.5%), n-butanol, tetrabutylammonium iodide (TBAI, 99%), acetone (95%), toluene (95%), n-Hexane (99%), methanol (98%), ethanol (99.7%), n-butanol (99.5%), KOH (85%), 1,2-ethanedithiol (98%), acetonitrile (99%). All chemicals were used as received without further purification.

Preparation of Mg-doped ZnO (MZO) precursor: the precursor was prepared by mixing 785 mg zinc acetate dihydrate, 97.7 mg magnesium acetate tetrahydrate, 4.8 mL 2-methoxyethanol, and 0.2 mL ethanolamine together and stirring under N_2_ flow at 50 °C for 24 h. The precursor was then cooled down to room temperature and sealed in glass bottles.

Synthesis of ZnO nanoparticles: the ZnO nanoparticles were synthesized according to the literature [16] with a modified recipe. An amount of 0.9788 g of zinc acetate dihydrate was added to 42 mL methanol in a three-neck flask. The mixtures were heated to about 60 °C under N_2_ flow. Then 0.469 g KOH was dissolved in 22 mL methanol and added dropwise to the solution. The resulted mixture was continuously stirred for 1.5 h at 62 °C and cooled down to room temperature. Finally, the ZnO NCs were collected by centrifuging and washing the mixture with methanol twice and dissolved into n-butanol with concentrations of 2.5–20 mg/mL.

Synthesis of PbS CQDs: the synthesis method can be found in the literature [16,17]. In a typical process, 0.47 g of PbO, 1.35 mL of oleic acid, and 12.7 mL of ODE were mixed and loaded into a 100 mL three-neck flask, and then degassed at 100 °C for 2 h under vacuum and transferred to N_2_ flow. The mixture was heated up to 130 °C. Two hundred and ten microliters of (TMS)_2_S were dissolved in 6.4 mL ODE and the mixture was rapidly injected into the solution in the three-neck flask. After 20 s, the reaction was quenched by soaking the flask in an ice bath. The PbS CQDs were then purified by centrifuging in acetone, toluene, and ethanol in sequence. Finally, the PbS CQDs were dispersed into n-hexane at a concentration of 40 mg/mL.

## 3. Results and Discussion

**Figure 1:**
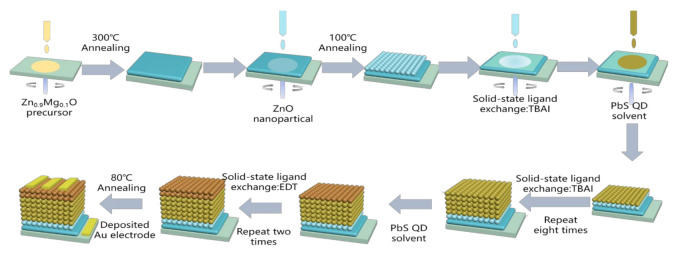
A schematic of PbS colloidal quantum dot (CQD) solar cell fabrication process under ambient conditions using layer-by-layer solution process.

The PbS CQD solar cells with an inverted structure of ITO/MZO/ZnO NC (*w/o*)/PbS CQDs/Au were fabricated via a layer-by-layer solution process at room temperature under ambient conditions, as shown in Figure 1. Firstly, the ITO substrates were cleaned by acetone and treated by O_2_ plasma for 10 min before the deposition of the active layer. The MZO thin film was prepared by spin-coating the MZO precursor on the ITO substrate at 3000 rpm for 20 s under ambient conditions, and annealed at 300 °C for 30 min on a hotplate. A thin layer of ZnO NC film was obtained by spin-coating the ZnO NC solution (with concentrations ranging from 2.5 mg/mL to 20 mg/mL) on the ITO/MZO substrate at 3000 rpm for 20 s. The ITO/MZO/ZnO NC samples were then annealed at 100 °C for 5 min to eliminate any organic solvents. Since the size of ZnO NCs is similar to that of PbS CQDs, we can decrease the misfit dislocations caused by the large lattice mismatch between the MZO and PbS CQDs, and obtain low dangling bond densities at the interface with the insertion of the ZnO NC layer. Upon this ITO/MZO/ZnO NC substrate, eight layers of TBAI-treated PbS CQDs and two layers of EDT-treated PbS CQDs were deposited in sequence. This whole device fabrication process can be found in the literature [16]. During the device fabrication, oleic acid (OA) capped PbS CQDs was replaced by TBAI or EDT, and the treatment does not change the absorption of the PbS CQD thin film but reduces the distance between PbS CQDs and the Fermi energy level. The final products were stored in air overnight for further oxidation and sequentially annealed at 80 °C for 10 min. Finally, a 100 nm thick Au layer was deposited on the ITO/MZO/ZnO NC/PbS CQDs by vacuum evaporation through a shadow mask that defined an active area of 5.70 mm^2^ (as shown in Figure S1).

**Figure 2:**
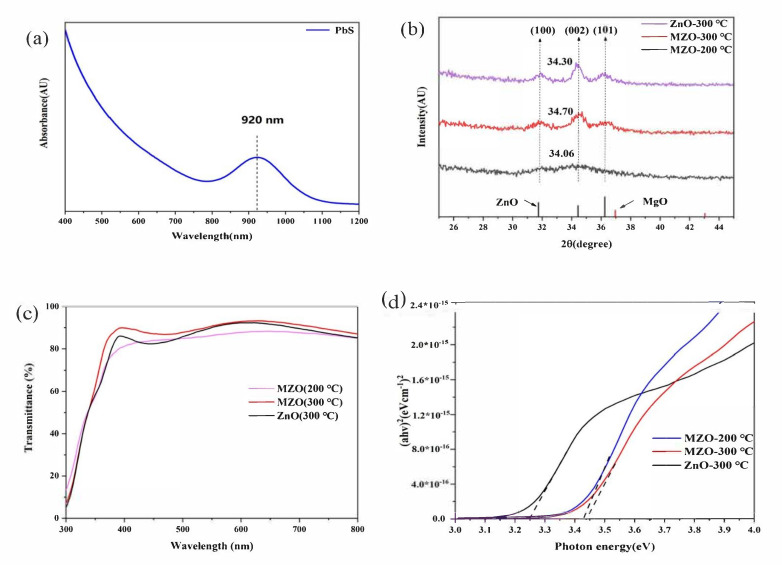
(**a**) Absorbance spectra of PbS CQDs for all devices listed here. (**b**) XRD pattern of ZnO and Mg-doped ZnO (MZO) thin films. (**c**) The transmission spectra of MZO with low annealing temperature (200 °C) and high annealing temperature (300 °C) and undoped ZnO thin film annealed at 300 °C. (**d**) The plots of (*ahv*)^2^ versus photon energy for MZO and ZnO thin films.

The UV absorption spectrum of PbS CQDs is presented in Figure 2a. It was clear that the absorption peak was located at 920 nm, corresponding to a bandgap of 1.35 eV. An X-ray diffraction (XRD) test was performed to investigate the crystal structure of the as-prepared MZO films. As shown in Figure 2b, when annealing at a low temperature, no peaks can be detected from the curve, which implies the formation of an amorphous structure under this treatment condition, which is consistent with results reported before [31]. As for the samples annealed at a higher temperature (300 °C), we could find diffraction peaks located at 31.9°, 34.3°, and 36.2°, corresponding to the (100), (002), and (101) facets of wurtzite ZnO (31.75°, 34.44°, 36.25°). It is evident that there is a preferred growth orientation along the c-axis of ZnO, as the (002) peak is the strongest. Compared to the ZnO film, the (002) peak of the MZO is shifted to a larger angle, which is due to the substitution of Zn^2+^ by small Mg^2+^, leading to a reduced crystal lattice parameter [21,22,31]. The transmission spectra of the MZO (ITO/MZO) treated with different annealing temperatures and the ZnO thin film are shown in Figure 2c. The MZO thin film annealed at 300 °C can block the light with a wavelength shorter than ~400 nm and was almost transparent for wavelengths >400 nm. In other words, MZO thin films are nearly transparent for visible light. At a lower annealing temperature (200 °C), the transmission is significantly lower than that of a higher annealing temperature (300 °C), which may be a consequence of the incomplete crystallization of MZO. From the plots of (*ahv*)^2^ versus the photon energy (Figure 2d), all the curves have a linear onset, indicating the existence of a direct bandgap. By extrapolating the linear region of these curves to intersection with the *x*-axis, we could determine that the bandgaps for the MZO thin film with different annealing temperatures were 3.43 eV and 3.44 eV, respectively, while this value was 3.25 eV for the ZnO thin film.

**Figure 3:**
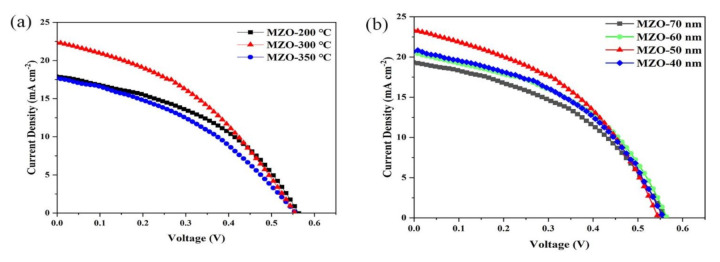
*J*-*V* characteristics of ITO/Mg-doped ZnO (MZO)/PbS CQDs/Au devices with (**a**) different annealing temperatures and (**b**) MZO thicknesses.

The MZO is a good ETL for PbS CQD solar cells due to the high transparency and high conduction band. To ascertain the optimal temperature and thickness of the PbS CQD active layer and MZO ETL for solar cells, devices with an inverted structure of ITO/MZO/PbS/Au were fabricated by depositing six PbS CQD layers on MZO film. A control device with ZnO ETL was also fabricated with the same conditions. It is obvious that an appropriate annealing temperature and thickness of MZO are necessary to obtain high-quality ETL thin films [31]. The current density–voltage characterization under dark conditions or 100 mW cm^−2^ (AM 1.5G) illumination (the irradiation area is 100 cm^2^) with a solar simulator (XES-40S1, SAN-EI electric) was carried out on a computer-controlled Keithley 2400 Source Meter system in ambient conditions. The *J*-*V* curves of PbS CQD solar cells with different annealing temperatures and thicknesses are presented in Figure 3a,b, and the photovoltaic performance is summarized in Table 1. To figure out the effects of annealing temperature on CQD device performance, the thickness of MZO is fixed at 50 nm for all cases. The devices with MZO annealed at a temperature of 300 °C showed the highest efficiency of 4.94% with a short circuit current density (*J*_sc_) of 22.44 mA/cm^2^, an open circuit voltage (*V*_oc_) of 0.55 V, and a fill factor (FF) of 39.75%. On the other hand, the devices annealed at a low temperature exhibited the lowest PCE (4.32%) due to the reduction of *J*_sc_. Increasing the annealing temperature further to over 350 °C also leads to a decrease in device performance because of the increase in series resistance, which has been confirmed previously [38]. We further investigated the effect of MZO thickness on the CQD device performance. In this case, the annealing temperature for MZO layers in the device is set to be 300 °C, while the thickness of the MZO layers is varied by changing the rate of spin-coating and the concentration of the MZO precursor. As shown in Figure 3b, the champion devices with 50 nm MZO showed the highest efficiency of 5.52%, with a short circuit current density (*J*_sc_) of 23.37 mA/cm^2^, an open circuit voltage (*V*_oc_) of 0.54 V, and a fill factor (FF) of 43.47%. The devices with an MZO thickness greater or less than 50 nm showed poor performance. As a result, we adopt 300 °C as the annealing temperature and 50 nm MZO for all the devices discussed below.

**Figure 4:**
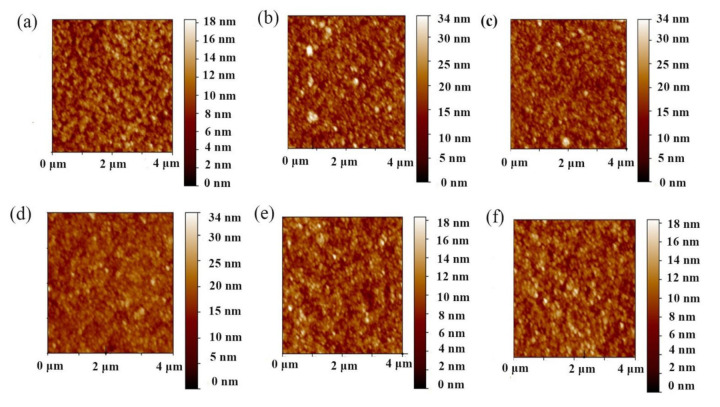
Atomic force microscopy (AFM) images of ITO/Mg-doped ZnO (MZO)/ZnO nanocrystals (NCs) (prepared by spin-casting ZnO NC solution onto the ITO/MZO substrate with different NC concentrations) with different thicknesses of ZnO NCs of (**a**) 0, (**b**) 10, (**c**) 15, (**d**) 20, (**e**) 30, and (**f**) 34 nm.

Since the high conduction band value of the MZO layer may prevent electrons being extracted from the active layer and lead to local carrier recombination, we introduced a thin layer of ZnO NC (~10–34 nm) between the MZO and PbS CQDs. To deposit ZnO NC film on ITO/MZO substrates, ZnO NC solution (ZnO NCs dissolved in n-butanol) with different concentrations (the thicknesses of the corresponding ZnO NC layers were 10 nm, 15 nm, 20 nm, 30 nm, 34 nm in 2.5 mg/mL, 5 mg/mL, 10 mg/mL, 15 mg/mL, 20 mg/mL, respectively) was spin-coated onto the substrates and annealed at 100 °C to remove any organic solvents. Atomic force microscopy (AFM) was used to characterize the surface morphology of ITO/MZO/ZnO NC samples with different thicknesses of ZnO NCs. As shown in Figure 4a–f, all samples showed very smooth surfaces with root mean square (RMS) values below 4 nm (the corresponding RMS values are 2.01 nm, 3.61 nm, 3.17 nm, 2.43 nm, 1.81 nm, and 1.82 nm). It was noted that the RMS values for 10 and 15 nm thick ZnO NC layers are 3.61 and 3.17 nm, respectively, significantly higher than other ZnO NC samples, which may be due to the insufficiency of ZnO NCs to fully cover the substrate at a low ZnO NC concentration. The smoothness of the ZnO NC surface is essential to promote the physical contact conditions between ZnO NCs and PbS CQDs and reduce interfacial recombination, and to improve device performance.

**Figure 5:**
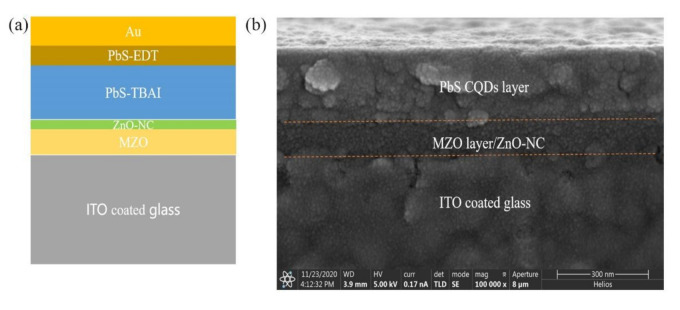

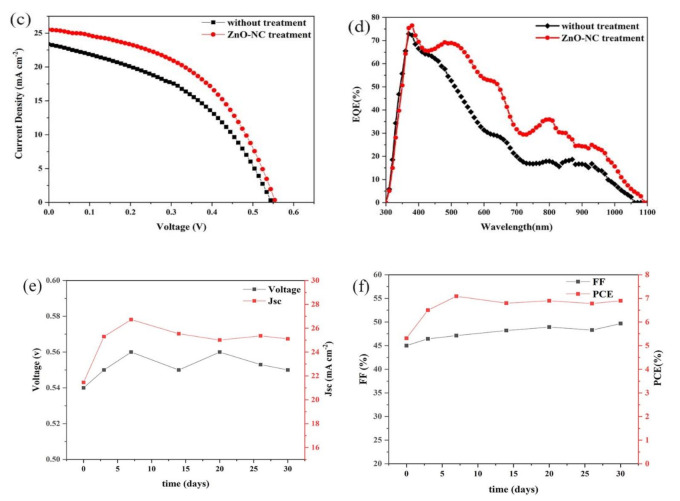
(**a**) The device structure of CQD solar cells, (**b**) cross-section SEM image of ITO/Mg-doped ZnO(MZO)/ZnO NC/PbS CQD thin film. (**c**) The *J-V* curves for optimized PbS CQD solar cells with and without ZnO NCs, and corresponding (**d**) EQE spectra. Stability test of CQD solar cells (**e**) *V_oc_* and *Jsc* and (**f**) FF and PCE; devices are stored under ambient conditions and all devices are encapsulated.

Now let us focus on the influence of the introduction of the ZnO NC thin film with different thicknesses on photovoltaic device performance. As we have just discussed, a ZnO NC thin film with an appropriate thickness is essential to eliminate interface defects and improve device performance. Figure 5a,b show the device structure and cross-section scanning electron microscopic (SEM) images of PbS CQD solar cells. From the SEM images, one can see that the ETLs (~70 nm) and PbS CQD (~300 nm) active layers are compact and there are no pinholes in a large area. The energy dispersive X-ray spectroscopy (EDS) spectrum presented in Figure S2 shows that the ratio between Pb and S is near 1:1, indicating the total conversion of S to PbS during the reaction. For this part of the research, the thickness of ZnO NC film was varied from 0 to 34 nm, whereas the thicknesses of the MZO and PbS CQD films were fixed at 50 and 300 nm, respectively, with the device structures of ITO/MZO/ZnO NC/PbS/Au. In other words, all the experimental parameters were fixed except the thickness of ZnO NCs. The PCEs with and without ZnO NCs are presented in Figure 5c. The *J*-*V* curves for different ZnO NC thicknesses under light are presented in Figure S2 and the dark *J-V* curves are presented in Figure S3. The detailed photovoltaic parameters are summarized in Table 2. It should be noted that the PCEs of PbS CQD solar cells increase with ZnO NCs from 15 to 20 nm, and experience a degradation as the thickness of the ZnO NC layer exceeds 20 nm. The optimal PCE is obtained in the 15 nm ZnO NC device, which shows the following merits: a *J*_sc_ of 26.72 mA/cm^2^, a *V*_oc_ of 0.56V, an FF of 47.12%, and a PCE of 7.09%. Meanwhile, the control device with a single MZO as an ETL exhibits a *V*_oc_ of 0.54 V, a *J*_sc_ of 23.36 mA/cm^2^, and an FF of 43.47%, resulting in a PCE of 5.52%. As a result, the optimal PCE value is 30% higher than that of the control device. For a device with a ZnO NC layer thickness of less than 15 nm, it is anticipated that interface recombination will occur because of the inadequate coverage of the MZO film. Moreover, when the ZnO NC layer is too thick, the series resistance will increase, which leads to low FF and PCE. The external quantum efficiency (EQE) spectra shown in Figure 5d provide insights for understanding how the *J*_sc_ of the devices was affected by the ZnO NC layer. In comparison to a device without ZnO NCs, the EQE spectrum of the ZnO NC device exhibits a high carrier extraction rate in the wavelength range from 400 to 900 nm, suggesting an enhanced carrier collection with the help of a ZnO NC interlayer since the interface defects can be effectively eliminated. The integrated current densities of 25 mA/cm^2^ and 22 mA/cm^2^ are obtained from EQE curves, which are consistent with our *J*-*V* measurement (Figure 5c). We noted that the FF of optimized PbS CQD solar cells is less than 50%, which implies that high series resistance and low parallel resistance existed in CQD solar cells. We speculate that although the introduction of ZnO NCs improves the FF and PCE of PbS CQD solar cells, carrier recombination will still occur in the active layer and the ZnO/PbS interface as the devices are fabricated under ambient conditions. The experimental conditions, such as humidity, temperature, and cleanliness, should be well controlled to further improve the performance of PbS CQD solar cells. It should be pointed out that the device stability is one of the major issues for PbS CQD solar cells. We examined the stability of NC solar cells by testing the device performance preserved under ambient conditions at intervals of several days over a relatively long period. In our CQD solar cell configuration, the stability is mainly related to the quality of the PbS CQD/ZnO NC interface, the PbS CQD active layer, and the contact electrode. As shown in Figure 5e,f, the PCE, *J*_sc_, and *V*_oc_ reach their maximum value at day 7, which then remained almost unchanged after preservation under ambient conditions for 30 days. The increase in device performance after 7 days may be due to the reduced interface recombination between CQDs and the CQD/Au interface, which was also confirmed in the literature [39]. The device with an MZO/ZnO NC ETL maintained 97.7% of its maximum PCE after being placed in ambient conditions for such a period. We speculate that the introduction of a ZnO NC layer might also benefit the stability of CQD solar cells.

**Figure 6:**
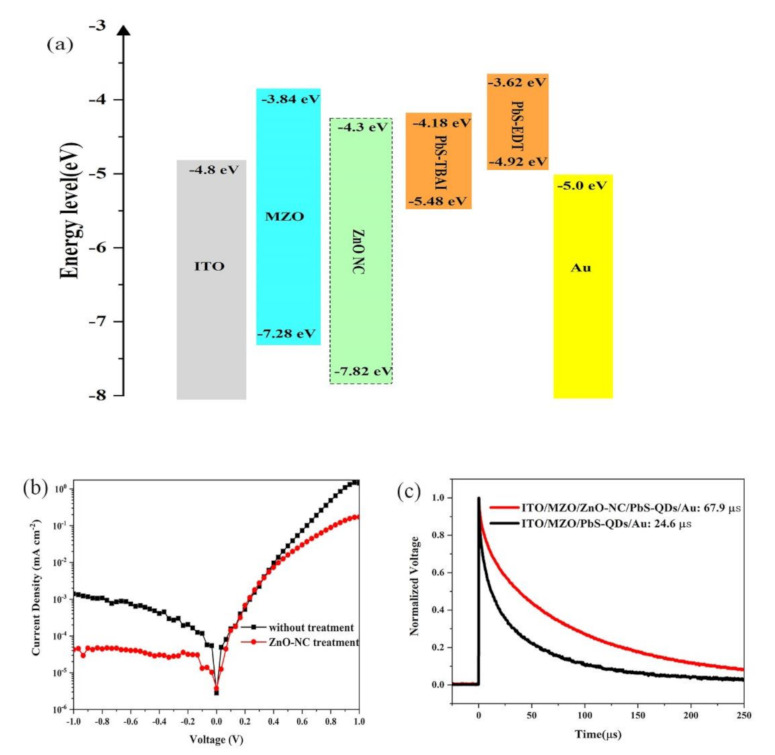
(**a**) The band alignment of PbS CQD solar cells with inverted structure of ITO/Mg-doped ZnO (MZO)/ZnO NC/PbS-TBAI/PbS-EDT/Au. (**b**) Dark *J-V* curves of PbS CQD solar cells with/without ZnO NC layer. (**c**) Transient photovoltage (TPV) measurements of PbS solar cells with/without ZnO NC layer.

To understand the influence of ZnO decoration on the PbS CQD device performance comprehensively, we measured the work function of the MZO and MZO/ZnO NC thin film by a Kelvin probe microscope. The Fermi levels of the MZO and MZO/ZnO NC are 4.49 eV and 4.37 eV, respectively. It can be seen that the introduction of ZnO NCs increased the Fermi level of the MZO thin film. The band diagram of ITO, MZO, ZnO NCs, PbS CQDs, and Au is presented in Figure 6a. As expected, the introduction of ZnO NCs increased the Fermi level, which is beneficial to the electron injection from CQDs to the ETL. Moreover, as the conduction band of ZnO NCs is almost the same as that of PbS CQDs, the small energy barrier at the interface will decrease carrier recombination. We measured the *J*-*V* characteristic of our device under dark conditions. As shown in Figure 6b, the current at the reversed bias for the device with a ZnO NC interlayer is almost one order lower than that of the device without a ZnO NC interlayer. The low leakage current implies that the ZnO NC interlayer can decrease the PbS CQDs/MZO interface defects and restrain carrier recombination, resulting in a significant improvement in device performance. To further illustrate the effects of the ZnO NC interlayer on the recombination process of PbS CQD solar cells, the transient photovoltage (TPV) is used to measure the charge recombination in PbS CQD solar cells with/without a ZnO NC interlayer. In this measurement, a steady state equilibrium is obtained when the NC solar cells are placed under a white light bias and additional charges are generated by applying another weak laser pulse. As shown in Figure 6c, the charge recombination is characterized by tracking the transient voltage associated with the perturbations in the charge population. The charge recombination times for NC devices with/without a ZnO NC interlayer are 67.9 μs and 24.6 μs, respectively, which imply a lower charge recombination rate in the ZnO NC interlayer device compared to devices without a ZnO NC interlayer.

## 4. Conclusions

In conclusion, we have fabricated efficient PbS CQD/ZnO heterojunction solar cells by a simple layer-by-layer solution process at ambient conditions. The introduction of an MZO window layer and a thin interfacial layer of ZnO NCs between the PbS CQDs and MZO could promote a band alignment condition and reduce interface defects. Compared to control devices, obvious improvements in *J*_sc_ and PCE are observed for devices with a ZnO NC interlayer. After carefully optimizing the fabrication parameters, we obtained a device with a PCE of 7.09%, which shows a 30% increase compared to the control device. Our work here provides a new way to improve the performance of PbS CQD solar cells and might throw light on the industrial application of CQD solar cells.

## Figures and Tables

**Table 1:** Summarized photovoltaic parameters from *J-V* curves (Figure 3a,b) of PbS CQD solar cells prepared under different conditions.

Annealing Temperature (°C)	MZO Layer Thickness (nm)	*V_oc_* (V)	*J_sc_* (mA/cm^2^)	FF (%)	PCE (%)
200	50	0.56	17.79	43.15	4.32
300	50	0.55	22.44	39.75	4.94
350	50	0.55	17.62	39.04	3.81
300	40	0.55	20.67	44.74	5.12
300	50	0.54	23.37	43.47	5.52
300	60	0.53	21.29	45.33	5.15
300	70	0.56	19.33	44.23	4.82

**Table 2:** Summarized photovoltaic parameters of PbS CQD solar cells prepared with different ZnO NC thicknesses (device structure: ITO/Mg-doped ZnO (MZO)/ZnO NC/PbS CQDs/Au).

ZnO NC Layer Thickness (nm)	*V_oc_* (V)	*J_sc_* (mA/cm^2^)	FF (%)	PCE (%)	$R_{s}$ (Ω cm^2^)	$R_{sh}$ (Ω cm^2^)
0	0.54	23.36	43.47	5.52	7.98	73.59
10	0.54	22.87	40.02	4.97	10.61	71.82
15	0.56	26.72	47.12	7.09	6.59	134.51
20	0.56	25.01	48.9	6.9	5.56	129.83
30	0.53	24.70	44.40	5.85	7.09	88.293
34	0.56	23.49	47.43	6.09	6.97	80.028

*R_s_*: Series resistance; *R_sh_*: parallel resistance.

## Supplementary Materials

The following are available online at https://www.mdpi.com/2079-4991/11/1/219/s1, Figure S1: The photo of a real PbS CQD solar cell. In the cathode part, the MZO/ZnO NC/PbS active layer has been scraped away before Au deposition. The active area is 0.057 cm2 for each device. Figure S2: SEM image and energy dispersive X-ray spectroscopy of ITO/MZO/ZnO-NC/PbS CQDs, Figure S3: J-V curves for PbS CQD solar cells with different ZnO NC layer thicknesses, Figure S4: dark J-V curves for PbS CQD solar cells with different ZnO NC layer thicknesses.
