# Supplementary material for: Efficient PbS Quantum Dot Solar Cells with Both Mg-Doped ZnO Window Layer and ZnO Nanocrystal Interface Passivation Layer
Source: Nanomaterials (Basel). 2021 Jan 15;11(1):219. doi: 10.3390/nano11010219 (PMC7830923; doi:10.3390/nano11010219)
Supplement: Supplementary file 1 [file nanomaterials-11-00219-s001.pdf]

Supplementary Materials

Efficient PbS Quantum Dot Solar Cells with Both Mg-Doped ZnO Window Layer and ZnO Nanocrystal Interface Passivation Layer

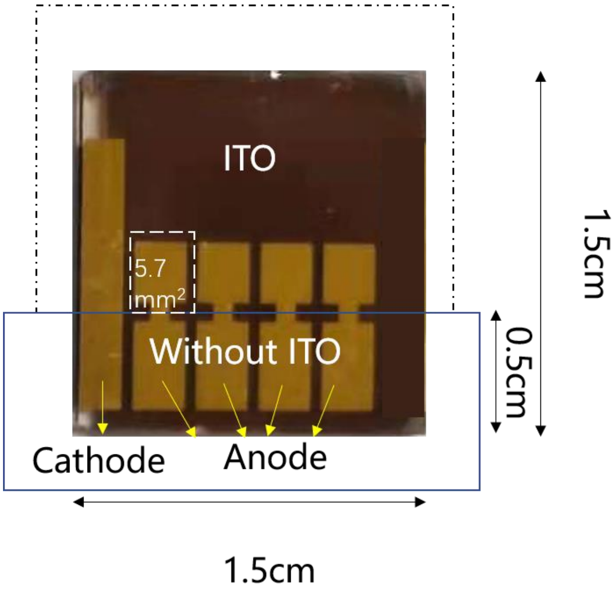

Figure S1. The photo of a real PbS CQDs solar cells, in the part of cathode, the MZO/ZnO NC/PbS active layer has been scraped away before Au deposition. The active area is 0.057 cm² for each device.

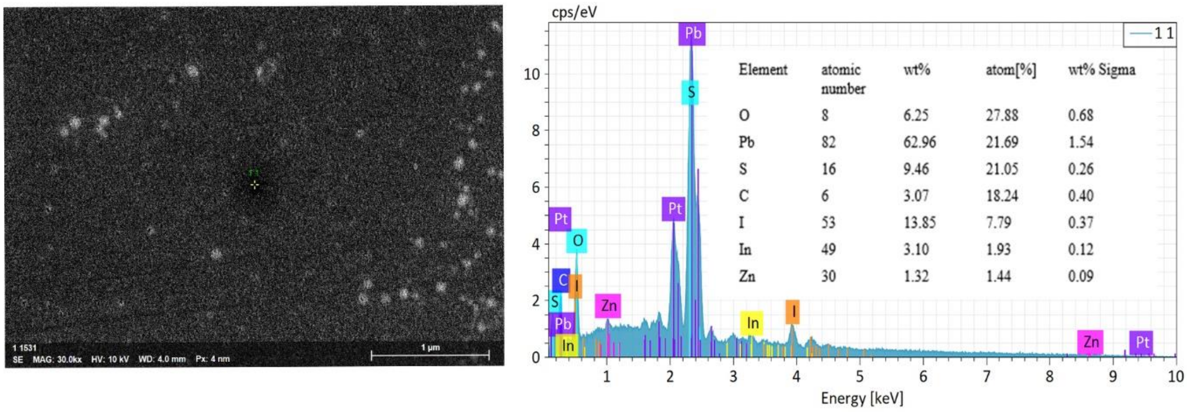

Figure S2. SEM image and Energy dispersive X-ray Spectroscopy of ITO/MZO/ZnO-NC/PbS CQDs.

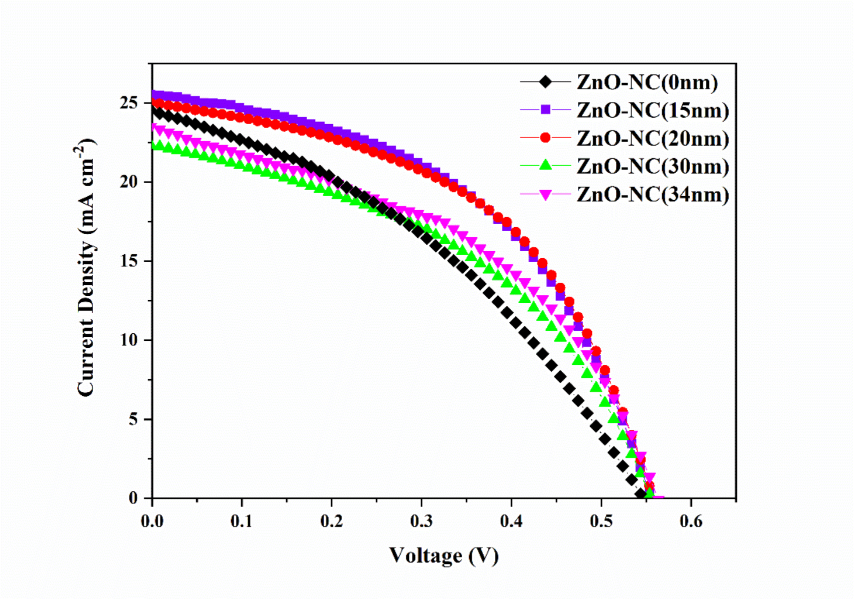

Figure S3. *J-V* curves for PbS CQDs solar cells with different ZnO-NC layer thickness.

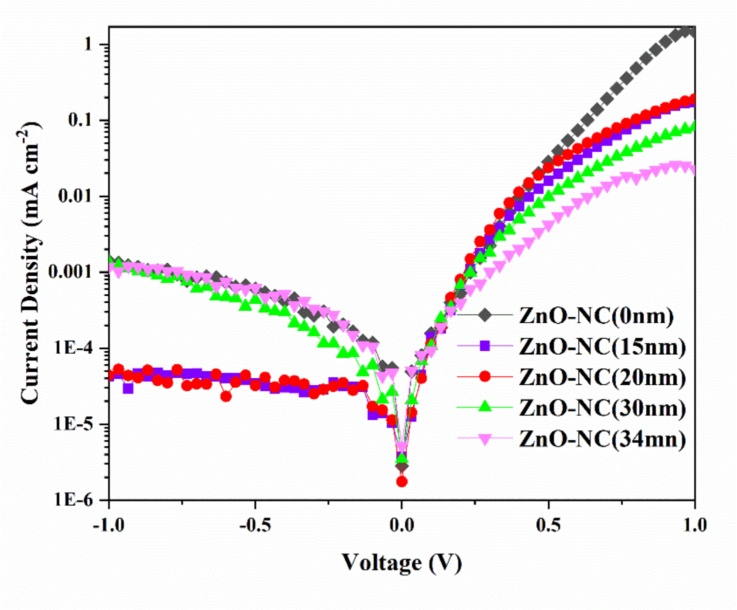

Figure S4. dark *J-V* curves for PbS CQDs solar cells with different ZnO-NC layer thickness.
